# Coping strategies of stress and its associated factors among breast cancer patients in Tikur Anbesa specialized hospital, Ethiopia: Institution-based *cross-sectional study*

**DOI:** 10.1186/s12905-022-01792-0

**Published:** 2022-06-24

**Authors:** Bethlehem Assefa Kelkil, Niguse Tadele Atnafu, Negalign Getahun Dinegde, Mulugeta Wassie

**Affiliations:** 1 Department of Medical Nursing, School of Nursing, College of Medicine and Health Sciences, University of Gondar, Gondar, Ethiopia; 2 Departments of Nursing, School of Nursing and Midwifery, College of Health Science, Addis Ababa University, Addis Ababa, Ethiopia

**Keywords:** Coping strategies, Stress, Breast cancer, Tikur Anbesa specialized hospital

## Abstract

**Background:**

Diagnosed with breast malignancy can be stressful, affecting several domains of life, affecting physical, emotional, and spiritual well-being that can lead to stress. To adapt to stress, the patient can use different coping methods. Therefore the objective of this research was to assess coping strategies for stress and its associated factors among breast cancer patients in Tikur Anbesa specialized hospital, Ethiopia.

**Methods and materials:**

The institution-based cross-sectional study was carried out among 272 study participants attending Tikur Anbessa specialized hospital from February to April 2020. The data was collected using a structured questionnaire and analyzed using Stata 4.2. Descriptive statistics was employed for data analysis and tables and figures were used to present the results. Binary logistic regression was used to identify variables that affected the outcome variables.

**Result:**

Majority (45.8%) of the study participants were in the age range 40–54 years. About 51.1% [95% CI (45.1–57.2)] of breast cancer patients have positive coping strategies to stress in the current study. About 64% solve stress through the Confrontive strategy and more than 73% of participants solve their problems by distancing. In self-controlling coping mechanisms, most participants do positive coping strategies. Having social support and taking only chemotherapy increased positive coping strategy but being single and time since diagnosis (1–3 years) increased negative coping.

**Conclusions:**

About 51% of breast cancer patients have a positive coping strategy. Since the majority of breast cancer patients in the current study experienced negative coping strategies, it is better to expand health education regarding stress coping strategies. In addition, it is better to link patients to clinical psychologists and organizations that aimed to social support to cancer patients.

## Background

Breast cancer causes psychological distress to the patients due to its incurable nature along with its reoccurrence than the diagnosis [1]. The psychological side effect of breast cancer treatments includes stress, anxiety, and depression [2]. Coping is an individual’s effort to control stress and adjust to the needs of added problems. Using different coping mechanisms relieves the effects of stress on an individual’s physical and psychological symptoms [3].

The study conducted in different countries showed that patients feel frightened by interventions, like chemo or radiotherapy, and they are concerned about losing their bodily integrity, independence, and social roles aside from the fear of dying [4, 5].

However, a study was done in Australia and Pennsylvania for women with breast cancer indicated that patients who used an active coping mechanism by engaging the importance of accepting their diagnosis and doing physical activities that provided social and emotional support, reading the Bible, sleeping as much as possible, and drinking alcohol [6–8].

About 68.6% of breast cancer patients who were on chemotherapy had good family support, 65.7% used the problem-focused coping, 30.4% used the emotion-focused coping strategy, 52.0% experienced moderate anxiety, and only 2.0% experienced severe anxiety in the study conducted in southern India [8–10]. Therefore, this study aimed to identify stress coping strategies and their associated factors for breast cancer patients in TASH.

## Methods and materials

### Study design, area, and period

An Institutional based cross-sectional study was conducted at the oncology center of TASH from February to April 2020. TASH is the biggest referral public hospital in Ethiopia established in 1972. It is the training center for health professionals with undergraduate and postgraduate programs [11]. The hospital is staffed with many health care professionals from various disciplines. The oncology unit in TASH is the national sole cancer referral center since established in 1997. It has an outpatient unit that gives service to new and follow-up patients and an in-patients unit (with 20 beds) [12]. On average, about 700 breast cancer patients have a follow-up annually. It is the only radiation therapy center in Ethiopia and the only cancer registry center for Addis Ababa city.

### Populations

The source population of this study was all breast cancer patients attending TASH oncology center whereas the study population was all breast cancer patients > 18 years visiting TASH oncology center during the study period and who fulfilled inclusion criteria. All breast cancer patients > 18 years and patients in the normal condition, sufficient to be able to make an interview were included. Those breast cancer patients who had unable to communicate were seriously sick during data collection and male breast cancer patients were excluded.

### Sample size determination

The required sample size was determined by using single population proportion formula taking 50% of the prevalence of positive stress coping among breast cancer patients since there is no previous study on the same topic in Ethiopia.

$${\text{n}} = \left( {{\raise0.7ex\hbox{${{\text{z}}\upalpha }$} \!\mathord{\left/ {\vphantom {{{\text{z}}\alpha } 2}}\right.\kern-\nulldelimiterspace} \!\lower0.7ex\hbox{$2$}}} \right)2\;{\text{*}}\;{\text{p}}\left( {1 - {\text{p}}} \right)/{\text{d}}2 2$$

where n = sample size, (α/2)^2^ =1.96, *P* = 50%, d = difference from the actual figures of source population (5%).

Therefore, the initial sample was 384.

Since the total population is less than 10,000 (on average 700 breast cancer patients on follow-up annually), the correction formula was used and the corrected sample size was 247, then including a 10% non-response rate = 247 × 10% + 2 47 = 272 was the final sample size.

### Operational definitions

#### Positive coping strategy

participants who scored above and equal to the mean (106.85) of the 52 coping activities.

#### Negative coping strategy

participants who scored below the mean(106.85) of 52 coping activities.

### Data collection instrument and quality assurance

Data was collected using a pre-tested and structured questionnaire. The questionnaire used to assess coping mechanisms contains 52 items that are adapted from Lazarus and Folk man [13]. The questionnaire is modified depending on the local situation and the research objective. It was first prepared in English and translated into the local language (Amharic) and translated back into English to check the consistency of data collection.

Pretesting of the questionnaire was conducted on 5% of the sample size within 2 weeks before data collection. All filled questionnaires were checked for completeness and consistency and necessary corrections were made accordingly.

### Data analysis

The data were coded, cleaned, checked, and entered into EPI info 7.2 then exported to Stata 4.2 for analysis. Descriptive statistics were computed and the results are presented using tables and figures. Frequencies were used to see the overall distribution of the study subjects with the variables under study. Binary logistic regression was used to identify independent variables (marital status, occupation, social support, treatment taken, time of diagnosis, residence, monthly income, education, and age) that can affect the outcome variable (stress coping strategies). Independent variables with a *p* value < 0.05 in multivariable analysis were declared as statistically significant with a 95% confidence level. And the adjusted odds ratio(AOR) was used as an indicator of the magnitude and direction of the association.

## Result

### Socio-demographic and clinical characteristics of breast cancer patients

A total of 264 breast cancer patients participated in this study making a response rate of 97%. Out of the total participants, 153 (58%) came from Addis Ababa city. The mean age of participants was 44.9 ± 12.81. More than half (56.8%) of the respondents were orthodox Christian followers and about 20% of the study participants can’t read and write.

Regarding marital status, 106 (40.2%) were married. The majority of the study participants were housewives and only 6.1% of them were farmers. More than 76% (201) took the combinations of breast cancer therapies. Nearly 70% (183) had social support, of which more than 80% were from their families and relatives. About 60% had fixed monthly income (Table 1).

**Table 1:** Socio demographic and clinical characteristics of breast cancer patients in Tikur Anbesa specialized hospital, Ethiopia, 2020 (n = 264)

Variables	Category	Frequency (n)	Percent (%)
Area of residence	Addis Ababa	153	58.0
	Out of Addis Ababa	111	42.0
Age	25–39 years	91	34.5
	40–54 years	121	45.8
	55–69 years	38	14.4
	70–84 years	14	5.3
Religion	Orthodox Christian	150	56.8
	Muslim	58	22.0
	Protestant	41	15.5
	Catholic	15	5.7
Level of education	No education	50	18.9
	Elementary completed	45	17.0
	High school completed	97	36.7
	Preparatory and above	72	27.3
Marital status	Single	77	29.2
	Married	106	40.2
	Divorced	44	16.7
	Widow	37	14.0
Occupation	Housewife	121	45.8
	Government employee	67	25.4
	Privet employee	39	14.8
	Farmer	16	6.1
	Merchant	21	8.0
Duration since diagnosis	Less than 1 years	62	23.5
	1–3 years	117	44.3
	3–5 years	62	23.5
	Greater than 5 years	23	8.7
Type of treatment	Chemotherapy	44	16.7
	Radiotherapy	11	4.2
	Surgery	8.0	3.0
	Combination of therapies	201	76.1
Social support	No	81	30.7
	Family and relatives	147	55.6
	Friends and acquaintances	23	8.7
	Nurse and physicians	13	4.9
Monthly income (ETB)	No fixed	103	39.1
	Less than 500	59	22.3
	500 and above	102	38.6

### Coping strategies of stress among breast cancer patients

Confronting, distancing,self-controlling, seeking social support, accepting responsibility, positive reappraisal, escape avoidance, and plan of full problem solving were the components of breast cancer patients’ stress coping strategies. Here a total of 52 lists of activities whether participants did or didn’t were asked.

The majority (62.1%) of the participants stood on their ground and fought for what they wanted and above 64% of them didn’t let it get to them while more than 73% of them coped by went along with fate. In the self-controlling coping, the highest percentages of participants do most of the activities for positive coping.

Regarding seeking social support, more than 81% talked to someone who could do something concrete about the problem and nearly 98% of them got professional help. Most participants didn’t do all activities of accepting responsibilities except only 76.5% told themselves things that helped them to feel better.

From escape avoidance coping activities, more than 72% hoped a miracle would happen, got away from it for a while, tried to rest or take a vacation, and tried to make themselves feel well by eating, drinking alcohol, smoking cigarette, and using medication and accepted it.

Nearly 60% bargained to get something positive from the situation, and 67% changed something so things would turn out all right (Table 2).

**Table 2:** Descriptive statistics of stress coping strategies of breast cancer patients in Tikur Anbesa specialized hospital, Ethiopia, 2020 (n = 264)

Coping strategies	Activities	Yes		No	
		n	%	N	%
Confrontive	I did something which I didn’t think would work, but at least I was doing something	140	53.0	124	47.0
	I let my feelings out somehow	81	30.7	183	69.3
	Stood my ground and fought for what I wanted	164	62.1	100	37.9
	I accepted the next best thing to what I wanted	92	34.8	172	65.2
	I took a big chance or did something very risky to solve the problem	169	64.0	95	36.0
	I tried to get the person responsible to change his/her mind	96	36.4	168	63.6
Distancing	Turned to work or substitute activity to take my mind off things	57	21.6	207	78.4
	I went along with fate; sometimes I just have bad luck	193	73.1	71	26.9
	I went on as if nothing had happened	50	18.9	214	81.1
	Looked for the silver lining, so to speak; tried to look on the bright side of things	78	29.5	186	70.5
	Didn’t let it get to me; refused to think too much about it	170	64.4	94	35.6
	Made light of the situation; refused to get too serious about it	109	41.3	155	58.7
Self-controlling	Tried not to burn my bridges, but leave things open somewhat	152	57.6	112	42.4
	I tried to keep my feelings to myself	192	72.7	72	27.3
	I tried not to act too hastily or follow my first hunch.	153	58.0	111	42.0
	I tried to keep my feelings from interfering with other things too much	165	62.5	99	37.5
	I went over in my mind what I would say or do	96	36.4	168	63.6
	I kept others from knowing how bad things were	90	34.1	174	65.9
Seeking social support	Talked to someone to find out more about the situation	86	32.6	178	67.4
	Accepted sympathy and understanding from someone	62	23.5	202	76.5
	I got professional help	258	97.7	6	2.3
	Talked to someone who could do something concrete about the problem	215	81.4	49	18.6
	I asked a relative or friend I respected for advice	101	38.3	163	61.7
	Talked to someone about how I was feeling	95	36	178	67.4
Accepting responsibility	Criticized or lectured myself	86	32.6	178	67.4
	I told myself things that helped me to feel better	202	76.5	62	23.5
	I made a promise to myself that things would be different next time	76	28.8	188	71.2
	I apologized or did something to make up	68	25.8	196	74.2
Positive reappraisal	Changed or grew as a person in a good way	175	66.3	89	33.7
	I came out of the experience better than when I went in.	134	50.8	130	49.2
	Found new faith	163	61.7	101	38.3
	Rediscovered what is important in life	163	61.7	101	38.3
	I prayed	262	99.2	2	0.8
	I was inspired to do something creative about the problem	51	19.3	213	80.7
	I changed something about myself	155	58.7	109	41.3
Escape avoidance coping activities	Hoped a miracle would happen	189	71.6	75	28.4
	Slept more than usual.	72	27.3	192	72.7
	Got away from it for a while; tried to rest or take a vacation	201	76.1	63	23.9
	Tried to make myself feel better by eating, drinking, smoking, using drugs or medication	208	78.8	56	21.2
	Avoided being with people in general	63	23.9	201	76.1
	Refused to believe that it had happened.	72	27.3	192	72.7
	Accepted it, since nothing could be done	209	79.2	55	20.8
	Wished that the situation would go away or somehow be over with	73	27.7	191	72.3
Plan full problem solving	Just concentrated on what I had to do next—the next step	71	26.9	193	73.1
	I tried to analyze the problem in order to understand it better	178	67.4	86	32.6
	I felt that time would make a difference—the only thing to do was to wait	101	38.3	163	61.7
	Bargained or compromised to get something positive from the situation	158	59.8	106	40.2
	Changed something so things would turn out all right	177	67.0	87	33.0
	I knew what had to be done, so I doubled my efforts to make things work	65	24.6	199	75.4
	Came up with a couple of different solutions to the problem	55	20.8	209	79.2
	I prepared myself for the worst	51	19.3	213	80.7
	I jogged or exercised	79	29.9	185	70.1

### Overall coping strategies for stress among breast cancer patients

The responses of each component of coping activities were summed. Then the mean of each component was computed and participants who scored above and equal to the mean were considered as having a positive coping strategy and those who scored below the mean value were considered as having a negative coping strategy.

In the Confrontive coping mechanisms of stress, 131 (49.62%) of breast cancer patients had positive coping, whereas, in self-controlling strategies, 127 (48.11%) had positive coping.

About 47% had positive coping strategies in the domains of social support. Nearly 51.5% accepted responsibility positively and similarly 51% positively reappraised stress.127 (48.11%) did escape avoidance coping and 142 (53.79%) had planned full problem-solving activities.

**Fig. 1:**
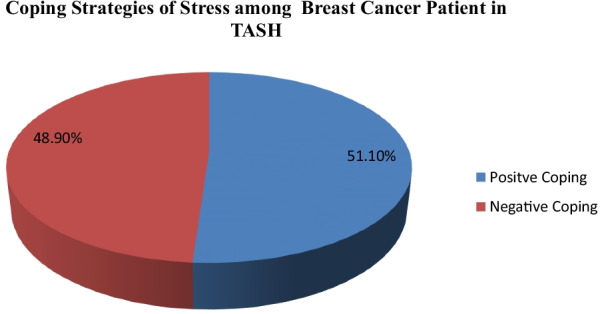
Stress coping strategy of breast cancer patients in Tikur Anbesa specialized hospital, Ethiopia, 2020 (n = 264)

The mean coping strategies score of stress among study participants was 106.85. The overall prevalence of positive coping strategies was found to be 51.1% [95% CI (45.1–57.2) in the current study (Fig. 1).

### Factors affecting stress coping strategies among breast cancer patients

Binary logistic regression was employed to identify independent variables that affect stress coping strategies for study participants.

Participants with a duration of breast cancer diagnosis in the range of 1–3 years coped with stress about 77% [AOR = 0.23, 95% CI (0.06–0.83)] lower than those who had a duration of greater than 5 years. Participants who had taken only chemotherapy coped with stress about 15times [AOR = 15.27, 95% CI (5.23–44.61)] more likely as compared with those taking the combined therapy (chemo, radiation, and surgery). Patients having social support challenged stress nearly 3 times [AOR = 3.36, 95% CI (1.66–6.75] more than their counterparts. Single patients coped stress about 55% [AOR = 0.45, 95% CI (0.23–0.85)] less than those who were married (Table 3).

**Table 3:** Results of factor analysis among breast cancer patients towards stress coping strategies in Tikur Anbesa specialized hospital, Ethiopia, 2020 (n = 264)

Variables	Categories	AOR	*P* value	95% CI
Age	25–39	0.29	0.192	0.05–1.84
	40–54	0.34	0.207	0.07–1.80
	55–69	0.39	0.265	0.07–0.06
	≥ 70	1.00	1.00	1.00
Education	Can’t read and write	1.00	0.992	0.39–0.53
	Primary	0.79	0.634	0.30–0.07
	Secondary	0.47	0.069	0.21–0.06
	College and above	1.00	1.00	1.00
Monthly income USD	Irregular monthly income	0.48	0.159	0.17–1.32
	< 9.77	1.78	0.196	0.74–0.31
	> 9.77	1.00	1.00	1.00
Residence	Addis Ababa	1.06	0.854	0.54–2.07
	Outside Addis Ababa	1.00	1.00	1.00
Time of diagnosis	< 1 year	0.27	0.105	0.05–1.32
	1–3 year	0.23	0.026*	0.06–0.83
	3–5 year	0.48	0.233	0.14–1.60
	> 5 years	1.00	1.00	1.00
Treatment taken	Chemotherapy	15.27	< 0.001*	5.23–44.61
	Radiotherapy	5.29	0.045*	1.04–26.95
	Surgery	5.94	0.058	0.94–37.61
	Combined	1.00	1.00	1.00
Social support	Yes	3.36	< 0.001*	1.66–6.75
	No	1.00	1.00	1.00
Occupation	House wife	1.01	0.989	0.26–3.78
	Employed	0.52	0.260	0.16–1.63
	Merchant	1.00	1.00	
Marital status	Single	0.45	0.014*	0.23–0.85
	Married	1.00	1.00	1.00

N/B: *variables significantly associated at *p* < 0.05; 1.00 = reference category

## Discussion

The current study has explored stress coping strategies and its associated factors among breast cancer patients attending in TASH. Overall, about 51% of breast cancer patients in the current study participated positively to stress coping activities. This is in line with studies conducted in Philadelphia [14]. However, the current study is not consistent compared with a study conducted in Australia [4]. This dissimilarity may be due to differences in lifestyle and socioeconomic characteristics of the study populations.

Around 47% of breast cancer patients had good social support. This finding is lower than the finding of a study conducted in Indonesia which is 68.6% [5]. This may be due to differences in the holistic palliative care delivery systems of the study settings in addition to the accessibility of organizations that can support patients.

About 62% of the participants stood on their ground and fought for what they wanted. The current finding is incongruent with the studies conducted in Berlin [15] and Taiwan [9]. This finding is not consistent compared with the Iranian study [16]. This may be due to differences in the cultural context and educational level of participants in respected study areas.

Nearly 64% of patients refused to think too much about it and more than 73% of them coped with stress by went along with fate in the distancing domain of coping strategies. This is consistent compared to the studies in Lebanon, British [1, 17], and Taiwan [9]. Yet significant differences have been seen compared to a study conducted in Egypt [18]. These might be cultural practice differences and social ideological differences.

In the self-controlling coping mechanisms, 36.4% went over in their mind what they would say, and 34.1% others not knew how bad things were. This result is almost consistent with the research done in Zambia [19]. Conversely, this finding is higher than the study done in Philadelphia [20].

Concerning looking for social support, greater than 81% of the participants communicated to somebody who could do things concrete for the problem, and close to 98% got professional help. The present finding is in agreement with the studies conducted in Indonesia [3], California, and America [21]. Nearly 72% of them hoped a miracle could have occurred. Near to 60% bargained for getting something positive in the condition and 67% altered something as things might be turned right. This is in line with the study done in Iran [22], and Lebanon [5].

Having social support and taking only chemotherapy positively affected the outcome while being single and diagnosed 1–3 years negatively affected stress coping.

Participants with social support coped with stress about 3.4 times more than their counterparts. The current result is in line with the studies conducted in the USA, China, and Iran [23–26]. The reason could be participants with social support accessibility might share their worries and stress with the supporters and get financial support for their treatment.

Patients diagnosed lately with breast cancer coped with their stress less than those diagnosed earlier. This finding is supported by a study conducted in Sweden [27]. This might be as patients diagnosed early could adapt to the situation and found alternative options too.

Patients who received only chemotherapy and radiation therapy exclusively coped with stress more than those who received the combined (chemoradiation and surgery) therapies. This result is supported by a study conducted in Sweden [27]. This could be as taking more than one type of therapy can increase the patient’s burden of treatment side effects that might affect positive coping strategies.

Another factor that affected stress coping in the current study was marital status. Single patients had coped with stress more negatively than married. This finding is supported by a review of the literature. The reason for this finding could be those single breast cancer patients would lose the opportunities of sharing bad feelings with their couples. Consequently, this might affect their stress coping negatively.

## Conclusions

About 51% of breast cancer patients have a positive coping strategy. Since the majority of breast cancer patients in the current study experienced negative coping strategies, it is better to expand health education regarding stress coping strategies. In addition, it is better to link patients to clinical psychologists and organizations that aimed to social support to cancer patients.

### Strength of the study

The strength of this study is its originality in providing information about coping strategies for stress and associated factors among breast cancer patients since there is insufficient information related to this area in the country at large.

## Limitations of the study

Since this study is a cross-sectional study, it shares the limitations of a cross-sectional study design. There could be also recalled bias on some variables. Some challenges during the data collection period as the patient flow of referral hospitals were decreased because of the COVID-19 pandemic were some of the constraints.

## Data Availability

The datasets generated and analyzed during the current study are not publicly available due to limitations of ethical approval involving the patient data and anonymity but are available from the corresponding author on reasonable request.

## Abbreviations

AAU: Addis Ababa University
AOR: Adjusted odd ratio
CI: Confidence interval
COR: Crude odd ratio
ETB: Ethiopian birr
SPSS: Statistical package for social science
TASH: Tikur Anbessa specialized hospital

## Declarations

For confidentiality, the names of the participant were not recorded instead codes were used. The study participants were informed about the right to refuse to join, ask any question or withdraw at any particular point during the data collection process without being frustrated.

All methods were performed in accordance with the relevant guidelines and regulations.
